# Efficient Fe_3_C-CF Cathode Catalyst Based on the Formation/Decomposition of Li_2−x_O_2_ for Li-O_2_ Batteries

**DOI:** 10.3390/molecules28145597

**Published:** 2023-07-24

**Authors:** Guanyu Yi, Gaoyang Li, Shuhuai Jiang, Guoliang Zhang, Liang Guo, Xiuqi Zhang, Zhongkui Zhao, Zhongping Zou, Hailong Ma, Xiaojiao Fu, Yan Liu, Feng Dang

**Affiliations:** 1 School of Materials Science and Engineering, Shandong Jianzhu University, Jinan 250101, China; yiguanyu@sdjzu.edu.cn (G.Y.); jiangshuhuai2022@163.com (S.J.); zouzhongping18@sdjzu.edu.cn (Z.Z.); mahailong@sdjzu.edu.cn (H.M.); fuxiaojiao18@sdjzu.edu.cn (X.F.); ly7623@sdjzu.edu.cn (Y.L.); 2 Key Laboratory for Liquid-Solid Structural Evolution and Processing of Materials (Ministry of Education), Shandong University, Jinan 250061, China; gaoyanglee@sdu.edu.cn (G.L.); guoliangzhang@mail.sdu.cn (G.Z.); 202014101@mail.sdu.edu.cn (L.G.); xiuqizhang@mail.sud.edu.cn (X.Z.)

**Keywords:** Fe_3_C-CF, Li-O_2_ batteries, Li_2−x_O_2_, DFT calculations

## Abstract

Lithium-oxygen batteries have attracted considerable attention in the past several years due to their ultra-high theoretical energy density. However, there are still many serious issues that must be addressed before considering practical applications, including the sluggish oxygen redox kinetics, the limited capacity far from the theoretical value, and the poor cycle stability. This study proposes a surface modification strategy that can enhance the catalytic activity by loading Fe_3_C particles on carbon fibers, and the microstructure of Fe_3_C particle-modified carbon fibers is studied by multiple materials characterization methods. Experiments and density functional theory (DFT) calculations show that the discharge products on the Fe_3_C carbon fiber (Fe_3_C-CF) cathode are mainly Li_2−x_O_2_. Fe_3_C-CF exhibits high catalytic ability based on its promotion of the formation/decomposition processes of Li_2−x_O_2_. Consequently, the well-designed electrode catalyst exhibits a large specific capacity of 17,653.1 mAh g^−1^ and an excellent cyclability of 263 cycles at a current of 200 mA g^−1^.

## 1. Introduction

Efficient-performance energy conversion and storage devices are eagerly pursued owing to the ever-increasing energy crisis. Of the various types of energy storage and conversion systems, Li-O_2_ batteries (LOBs) make it possible to store and release energy with high a theoretical energy density (~3500 Wh kg^−1^), which is nearly ten times higher than that of conventional Li-ion batteries [1,2,3,4,5]. In a typical non-aqueous lithium-oxygen battery system, the electrochemical reaction of the lithium-oxygen battery is ${2\mathrm{Li}}^{+}{+2e}^{-}{+O}_{2}\;⟷{\;\mathrm{Li}}_{2}O_{2}$ [6]. As the discharge product Li_2_O_2_ of the electrochemical reaction is an insulating product and does not decompose in the electrolyte [7,8,9], the battery reaction requires a complex solid–liquid–gas three-phase interface [10]. These negative factors lead to the actual capacity of the LOBs being much lower than their theoretical capacity, as well as high reaction overpotential and side reactions, which greatly deteriorate the electrochemical performance of the battery. The effective solution to enhance the electrochemical performances of LOBs is to select cathode catalysts with high activity that provide sufficient reaction sites to accelerate the oxygen reduction reaction (ORR)/oxygen evolution reaction (OER) processes, and large space for accommodating discharge products. 

Fortunately, the breakthrough of nanomaterial synthesis technology provided a solution for the limited electrochemical performance of LOBs. In particular, one-dimensional nanomaterials with a large porosity and a high specific surface area that facilitate O_2_ and electrolyte diffusion and a low electrical conductivity reduce the reaction energy barrier of the battery [11,12,13,14,15]. Notably, carbon nanotube materials modified with noble metal nanoparticles (Pt, Pd, and Au) have attracted much attention due to their distinctive structure and performance for excellent catalytic activity and electrical conductivity [16,17,18]. Even though noble metal-based catalysts exhibit significant improvements in the catalytic activity of the ORR and OER compared with other transition metals, their liability and expensive price limit their broad use in the field. Therefore, many other carbon-support catalysts, including transition metal, transition metal oxide [19,20,21,22,23,24], and transition metal carbide [25,26,27,28,29,30,31,32] electrocatalysts, are expected to be reliable substitutes because of their low cost, abundant resources, good conductivity, diverse valence states, and high catalytic efficiency. In this way, one- or two-dimensional nanomaterials modified with transition metals or their metal carbides, oxides, or nitrides are considered one of the most promising catalysts for commercialization. For example, CoO and Co nanoparticles decorated with N/O dual-doping carbon nanofibers prepared by electrospinning possessed an outstanding capacity of 8798.6 mAh g^−1^ and an excellent reversibility of more than 140 cycles at a fixed capacity of 600 mAh g^−1^ [12]. It is reported that CeO_2_ nanorods show the best electrocatalytic activity, owing to the high content of oxygen vacancies on their surface; the cathode could be cycled for 200 and 70 cycles under a limited specific capacity of 600 and 1000 mAh g^−1^, respectively [13]. 

Here, we propose a reasonable strategy to prepare evenly loaded Fe_3_C–carbon fiber composite materials with a mesoporous structure. Fe_3_C particles anchored in carbon fibers can not only effectively avoid the agglomeration and growth of particles, but also prevent the catalytic particles from falling off in the catalytic reaction. Density functional theory (DFT) calculations show that the strong adsorption of Li and O_2_ on the (121) crystal plane reduces the reaction energy barriers of the ORR and OER processes and achieves low OER overpotential through Li-PATH (delithium of Li_2_O_2_ and decomposition of LiO_2_), which is conducive to the formation and decomposition of Li_2−x_O_2_. Hence, the catalyst can maintain good catalytic performance and stability in the catalytic reaction and promote the electrode reaction efficiency of LOBs. Benefiting from its innovative and peculiar architecture with a large specific surface, mesoporous structure, plentiful dispersed active sites, and high electrical conductivity, the Fe_3_C-CF catalyst exhibited great electrochemical performance in terms of its large specific capacity, outstanding rate capability, and long cycle life.

## 2. Results and Discussion

### 2.1. Synthesis and Characterization

**Figure 1:**
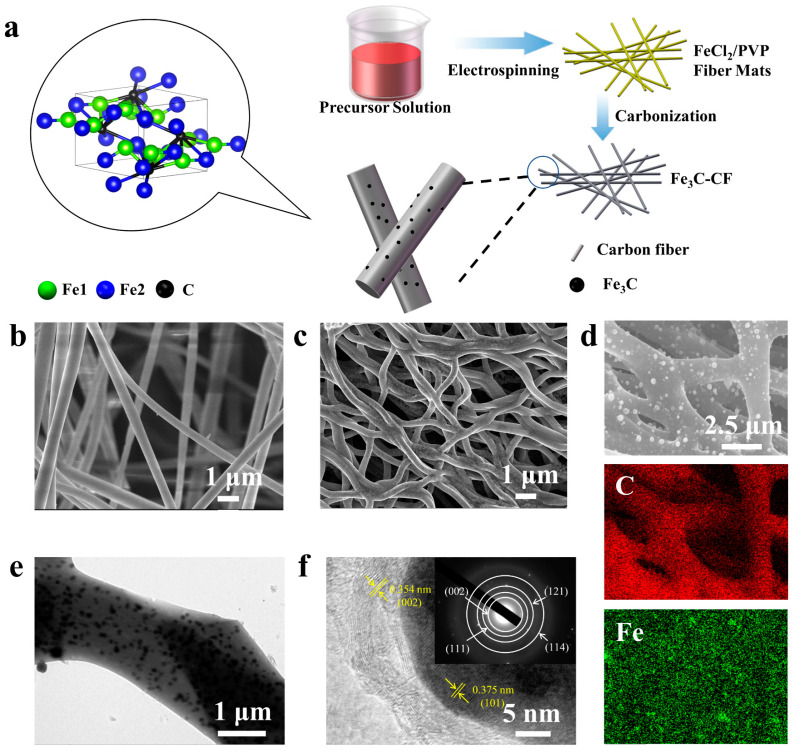
(**a**) Schematic illustration of Fe3C preparation; (**b**) SEM image of Fe3C-700 before carbonization; (**c**) SEM image of Fe3C-700 after carbonization; (**d**) SEM image and the corresponding EDS patterns of C and Fe in Fe3C-700; (**e**) TEM image of Fe3C-700; (**f**) HRTEM image of Fe3C-700 and with the corresponding SAED pattern inset.

As schematically illustrated in Figure 1a, the electrospinning strategy coupled with annealing was applied to fabricate the Fe_3_C-CF composites, which gives an overview of a typical setup. After electrochemical spinning, as shown in Figure 1b, the samples possess a typical fiber structure with a smooth surface and a diameter of 500 nm. During the carbonization process, polyvinyl pyrrolidone was transformed into the N-C structure via a pyrolysis reaction, accompanied by gas (CO_2_, H_2_O, and NH_3_) formation [33], and pores were introduced on the fiber surfaces. Simultaneously, the Fe-containing compound in the precursor reacts with graphite to generate Fe_3_C particles. Therefore, the Fe_3_C-CF composites were obtained. Notably, because of the higher temperature during annealing, the fibers of Fe_3_C-800 (Figure S1b) became more liable to bond and fracture than those of Fe_3_C-600 (Figure S1a) and Fe_3_C-700 (Figure 1c). 

As shown on the SEM images and energy-dispersive X-ray spectra (EDS) patterns in Figure 1c,d and Figure S2a,b, Fe_3_C particles are uniformly distributed in the composite fibers; the pictures indicate that the size of Fe_3_C particles increased with the rise in temperature. Furthermore, the TEM image (Figure 1e) also illustrates that a great quantity of particles were distributed uniformly on the fibers, whose average size was about 70~150 nm. Linear scanning experiments were also carried out on the Fe_3_C-CF, which showed a sharp spike from the Fe element as the scanning rays passed through the particles (Figure S3). In the high-resolution transmission electron microscopy (HRTEM) image (Figure 1f), the lattice spacing of the particles is 0.375 nm, corresponding to the (101) plane of Fe_3_C, while the lattice spacing of the fiber edge is 3.354 nm, corresponding to the (002) plane of carbon fiber. The selected area electron diffraction (SAED) pattern inside Figure 1f indicates that the diffraction rings are well indexed to the (002), (111), (121), and (114) planes of Fe_3_C. The above experimental results show that Fe_3_C particles are successfully prepared and uniformly loaded on carbon fibers.

**Figure 2:**
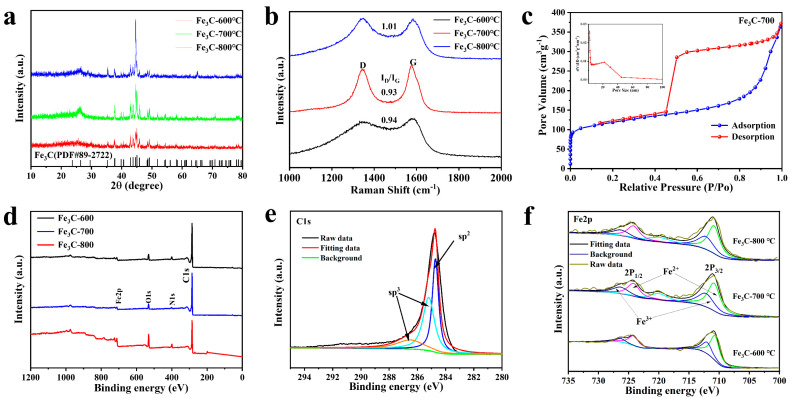
(**a**) X-ray diffraction (XRD) patterns of different Fe_3_C-CF samples; (**b**) Raman spectra of Fe_3_C-600, Fe_3_C-700, and Fe_3_C-800; (**c**) nitrogen adsorption/desorption isotherms, with the pore size distribution (inset) of Fe_3_C-700; (**d**) XPS survey spectra of Fe_3_C-600, Fe_3_C-700, and Fe_3_C-800; (**e**) XPS spectra (C1s) of Fe_3_C-700; (**f**) XPS spectra (Fe2p) of Fe_3_C-CF.

To verify the crystalline structure of the synthesized Fe_3_C-CF, typical powder X-ray diffraction (XRD) patterns are shown in Figure 2a. The three diffraction peaks at 2θ angles of around (43.75°, 44.59° and 45.01°) are matched to planes of Fe_3_C (ICSD PDF#89-2722), respectively. This result indicates that the FeCl_2_/PVP precursor has been completely transformed into Fe_3_C. The broad peak at around 26° is consistent with that of graphite [34,35,36], indicating the disordered graphitic structure of the fibers, while the graphitic carbon phase in Fe_3_C-700 showed the best crystallinity because of the highest peak at 26°. The graphitic carbon phase in Fe_3_C catalysts can enhance the electrical conductivity and durability of carbon, and, thus, stable ORR and OER active sites can be confined.

To investigate the degree of graphitization of carbon in Fe_3_C-CF, the Raman spectrum is shown in Figure 2b. The ratios of the G-band (1583 cm^−1^) to the D-band (1350 cm^−1^) were used to judge the degree of the graphitization [35,37,38]. The I_D_/I_G_ values for Fe_3_C-600, Fe_3_C-700, and Fe_3_C-800 were 0.94, 0.93, and 1.01, respectively, while the optimum graphitization of polymers like polyvinyl pyrrolidone or polyacrylonitrile generally occurs above 1000 °C, confirming that the Fe compound favors the graphitization of Fe_3_C-CF [39,40,41]. The lower I_D_/I_G_ value of Fe_3_C-700 indicated that 700 °C was more favorable to the growth of graphite microcrystals, resulting in better conductivity. Meanwhile, with the increase in carbonization temperature, a large amount of gas, such as H_2_O, CO_2_, and others, escaped, leading to a porous structure. To investigate the porous structure of Fe_3_C-CF samples, the specific surface area and the pore size distribution of Fe_3_C-CF were tested by nitrogen adsorption and desorption experiments (Figure 2c and Figure S4a–d). The isotherms with typical hysteresis loops can be classified as type IV, indicating the mesoporous structure of Fe_3_C-CF. Fe_3_C-600, Fe_3_C-700, and Fe_3_C-800 show a large specific surface area of 354.0, 387.6, and 314.2 m^2^ g^−1^, respectively, calculated by the BET method. From the above data, it can be seen that the specific surface area and graphitization degree of Fe_3_C-800 are reduced compared with Fe_3_C-700 due to the high temperature. A large specific surface area can not only provide enough space for the storage of discharge products to ensure the capacity of the battery, but it can also expose more active sites to improve catalytic performance. Furthermore, the pore sizes of Fe_3_C-700 are mainly around 3~40 nm, which are clearly higher than those of Fe_3_C-600 (3~20 nm) and Fe_3_C-800 (3~5 nm). The mesoporous structure of Fe_3_C-CF can accommodate the volume change in the electrochemical process, which is conducive to promoting electrolyte immersion, transporting oxygen and intermediate products, and providing three-phase reaction sites. It can be concluded that a proper temperature is useful for graphitization and obtaining a high specific surface area, which is beneficial to improving electrochemical performance.

During the electrochemical reaction of the battery, the change of valence state of Fe^2+^/Fe^3+^ is closely related to the catalytic activity of the material. The chemical composition and the valence state of Fe_3_C-CF were further characterized by XPS spectrum. As shown in Figure 2d, the total spectra of the synthesized Fe_3_C-CF samples displayed the core energy levels of Fe 2p, O1s, and C1s. The relative contents of C and Fe calculated from the survey spectra were 94.79/5.21, 98.33/1.67, and 98.42/1.58% for Fe_3_C-600, Fe_3_C-700, and Fe_3_C-800. The C1s spectrum can be fitted into three peaks, among which the peak at 284.72 eV is related to the Fe-C bond and comes from the sp^2^ hybrid carbon, while the peaks at 285.2 eV and 286.5 eV correspond to the sp^3^ hybrid C-C bond (Figure 2e) [12,30,35]. In the fine spectrum of Fe 2p (Figure 2f), the Fe 2p_3/2_ and Fe2p_1/2_ peaks can be fitted into two pairs of peaks, including Fe^2+^ (710.9 and 724.3 eV), Fe^3+^ (712.4 and 726.5 eV), and a satellite peak at around 720.1 eV [35,42], while the calculated ratio of Fe^2+^ to Fe^3+^ in the three samples is close to 1:1, providing a uniform distribution of valence states for catalytic reaction. In particular, there is no peak of pure iron (706.7 eV) in the spectrum of Fe 2p, which proves that the material prepared in this paper is pure Fe_3_C phase.

### 2.2. Electrochemical Performance of the Fe_3_C Cathode

**Figure 3:**
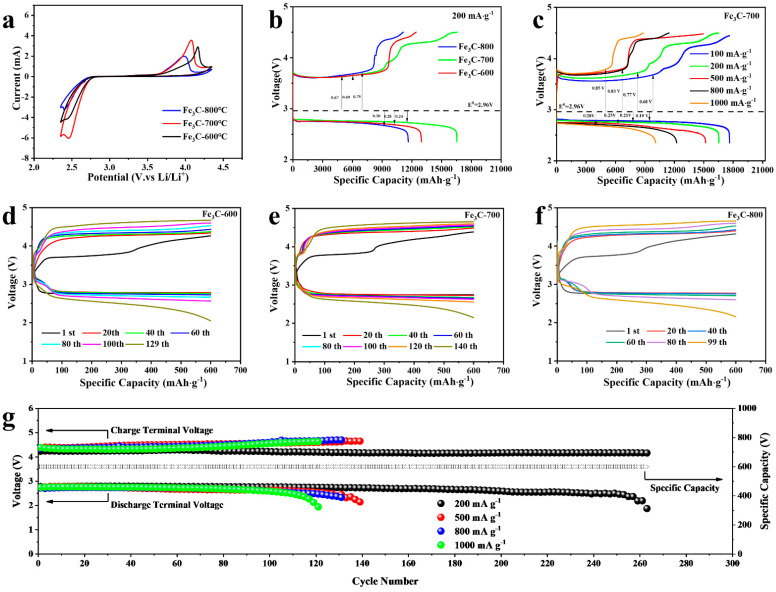
(**a**) Cyclic voltammetry curves (CV) for different cathodes at a scan rate of 0.15 mV s^−1^ within a voltage window of 2.35–4.35 V; (**b**) initial discharge/charge profiles of different catalytic electrodes at 2.35–4.5 V (**c**) Rate capability of Fe_3_C-700 electrode at different current densities; galvanostatic discharge/charge curves of (**d**) Fe_3_C-600, (**e**) Fe_3_C-700, and (**f**) Fe_3_C-800 within an upper-limited specific capacity of 600 mAh g^−1^ at a current density of 500 mA g^−1^; (**g**) comparison of galvanostatic discharge/charge cycles and corresponding terminal voltages of Fe_3_C-700 with an upper limited specific capacity of 600 mAh g^−1^ at a current density of 200 mA g^−1^, 500 mA g^−1^, 800 mA g^−1^ and 1000 mA g^−1^.

To investigate the electrochemical performance of the Fe_3_C-CF cathode, CR2032-type batteries with a lithium plate as the counter electrode were assembled. In Figure 3a, the reversibility and redox mechanism of the electrochemical reactions are studied by cyclic voltammetry (CV) with a scan rate of 0.15 mV s^−1^ at a voltage window of 2.35~4.35 V versus Li/Li^+^. The CV plots of the Fe_3_C-700 sample show both higher cathodic ORR onset potential (at 2.45 V) and lower anodic OER (at 4.07 V) with a larger integral area compared with other samples. As shown in Figure S5, electrochemical impedance spectroscopy (EIS) spectra were also collected before and after CV rest at open-circuit voltage, with the frequency ranging from 10^5^ to 0.01 Hz. The charge transfer resistance (R_ct_) can be evaluated by the diameter in the middle frequency range. Obviously, the R_ct_ of the Fe_3_C-700 cathode has a small value of 23.63 Ω compared with the Fe_3_C-600 cathode (38.94 Ω) and the Fe_3_C-800 cathode (27.13 Ω). In addition, the Fe_3_C-700 cathode also presented a slight increase after CV cycles, which contributed to the formation and incomplete decomposition of insulating discharge products, at 50.54 Ω. The CV and EIS results demonstrated that the Fe_3_C calcined at 700 °C possessed favorable reversibility and a large capacity as a cathode for LOBs. The first discharge and charge processes were carried out to evaluate the capacity performance of cathodes in LOBs. As illustrated in Figure 3b, at 200 mA g^−1^ within the limited voltage window of 2.35~4.5 V, the Fe_3_C-700 cathode presents a low overpotential of 0.24/0.67 V and a high discharge/charge specific capacity of 16,556.0/16,537.1 mAh g^−1^ with a columbic efficiency of 99.8%. However, those of Fe_3_C-600 and Fe_3_C-800 cathodes only delivered a discharge/charge specific capacity of 12,979.4/12,439.5 mAh g^−1^ and 11,646.6/11,148.5 mAh g^−1^ with a columbic efficiency of 95.8/95.7%, respectively. Regarding tge Fe_3_C-700 cathode, the discharge curves appear to be stable above 2.72 V, and there are two stages at a low platform of 3.64 V and a high platform around 4.25 V, which can be ascribed to two steps of decomposition of the discharge products. The amorphous Li_2−x_O_2_ decomposition first takes place at a low charge voltage for its high electroconductivity, while the insulting Li_2_O_2_ decomposes at a subsequent high charge voltage. For the Fe_3_C-700 cathode, a good rate capability is also achieved, as depicted in Figure 3c. As the current density increases, excellent discharge/charge performances of 17,531.1/17,640.7, 16,556.0/16,537.0, 15,203.9/149,72.7, 12,254.5/11,481.5, and 10,106.7/8848.2 mAh g^−1^ are obtained for the Fe_3_C-700 cathode with a slight polarization increase and capacity decline at 100, 200, 500, 800, and 1000 mA g^−1^, respectively. Further, selected discharge/charge profiles and the depicted cycle number associated with the terminal voltage of different cathodes at 500 mA g^−1^ with a cut-off capacity of 600 mAh g^−1^ were evaluated and are presented in Figure 3d, e, and f. It is clear that the Fe_3_C-700 cathode can run steadily for 140 cycles, while the Fe_3_C-600 and Fe_3_C-800 cathodes gradually fade away after 129 and 99 cycles. Moreover, the Fe_3_C-700 cathode exhibits an impressive cycle performance of 263 cycles (over 1500 h) at a low current density of 200 mA g^−1^ (Figure S6a). However, at a high current density of 800 and 1000 mA g^−1^, the Fe_3_C-700 cathode could also yield a stable long cycle life of 124 and 115 cycles, respectively (Figure 3g and Figure S6b,c).

### 2.3. Discharge/Charge Characteristics of Fe_3_C Cathode

**Figure 4:**
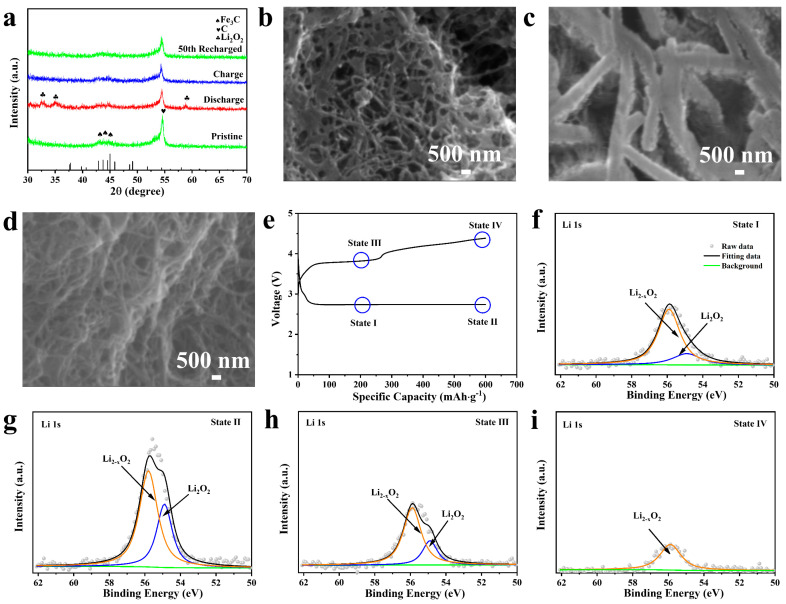
(**a**) XRD patterns at different discharge/charge stages; (**b**) SEM images of the Fe_3_C-700 cathode at the fresh stage; (**c**) the first cycle, full discharge; (**d**) the first cycle, full charge; (**e**) the first discharge–charge curve of the Fe_3_C-700 electrode at a current of 500 mA g^−1^ with an upper-limited capacity of 600 mAh g^−1^; high-resolution XPS spectra of the Li 1s core level of the Fe_3_C-700 electrode at different states corresponding to states (I~IV) in (**f**–**i**), respectively.

In order to reveal the kinetics and reaction mechanism of the Fe_3_C-700 cathode during the ORR/OER process, XRD, EIS, SEM, and ex situ XPS at various stages were conducted to investigate the structure and morphology of discharge products. As shown in Figure 4a, the XRD pattern of the first discharged Fe_3_C-700 cathode exhibited two characteristic peaks that corresponded to the discharge product Li_2_O_2_ (PDF#73-1640). After the 1st recharging and even after recharging for the 50th cycle, the peaks for Li_2_O_2_ disappeared and no other phases were formed. This indicated that the Fe_3_C-700 cathode could deliver excellent cycling reversibility with limited side reactions. The EIS plots of the Fe_3_C-700 cathode at different stages were recorded and are shown in Figure S7. R_ct_ increased from 23.63 Ω to 41.19 Ω after the first discharging, which could be caused by the deposition of insulated Li_2_O_2_ on the surface of the cathodes. It can be decreased to 38.94 Ω after the 1st charging, mainly due to the decomposition of Li_2_O_2_. Notably, the R_ct_ increase of the Fe_3_C-700 cathode after the 50th cycle is not obvious, indicating its good reversibility. The morphological revolution was included and discussed to support the above conclusion. As shown in Figure 4b, the Fe_3_C-700 cathode can be observed on the fresh cathode. After discharging to 600 mAh g^−1^, film-like discharge products are coated evenly on the Fe_3_C-700 cathode in Figure 4c. According to previous reports, the film-like products are most likely to be Li_2−x_O_2_, which is more conducive to disintegrating in the charging process. After recharging to the limited specific capacity (Figure 4d), the discharge products disappeared and the original morphologies were regained, similar to those of the pristine cathode of the Fe_3_C-700 cathode in Figure 4b. In order to reveal the characteristics of the Fe_3_C-700 cathode during the discharge and charge process with a fixed capacity of 600 mAh g^−1^ at a current density of 500 mA g^−1^, ex situ high-resolution XPS was conducted with selected key states, as shown in Figure 4e–i. When the discharge specific capacity reaches 200 mAh g^−1^ (State I in Figure 4f), two peaks can be obtained by fitting the high-resolution XPS spectrum of Li 1s. The peak that appeared at around 55.8 and 54.9 eV in the Li 1s profiles can be associated with the Li_2−x_O_2_ and Li_2_O_2_ discharge products [42,43]. The discharge products were mainly Li_2−x_O_2_ even after discharging to 600 mAh g^−1^ (State II in Figure 4g). After recharging to 200 mAh g^−1^ (State III in Figure 4h), it is obvious that the peak area ratio between Li_2−x_O_2_/ Li_2_O_2_ (76.8%) increased compared with that at State II (65.2%), denoting the preferential delithiation and facile decomposition of Li_2_O_2_ to form the Li_2−x_O_2_ intermediate at the initial stage of recharge. In the after-recharging process (Figure 4h), the intensity of the discharge products gradually decreases, indicating the decomposition of the discharge products. The existing small peak in the final state (State IV in Figure 4i) can be assigned to Li_2−x_O_2_, and the undecomposed products will gradually accumulate and passivate the Fe_3_C-700 cathode. In situ DEMS analysis offers reaction kinetics information for the Fe_3_C-700 cathode in the charging process by checking the released gas species. Figure S8a displays the DEMS result measured at 200 mA g^−1^ with a fixed capacity of 600 mAh g^−1^. The O_2_ evolution rate maintains a platform above the 2e^−^/O_2_ dotted line, demonstrating the decomposition of amorphous Li_2−x_O_2_. The small CO_2_ evolution at the end of the charge process might be caused by the corrosion of CNT or the side reaction of the electrolyte. After 50 cycles, XPS of C 1s spectra (Figure S8b) indicate that byproducts such as lithium carbonate are produced on the surface, which is due to accumulation during the recycling process [44].

### 2.4. Theoretical Calculations

**Figure 5:**
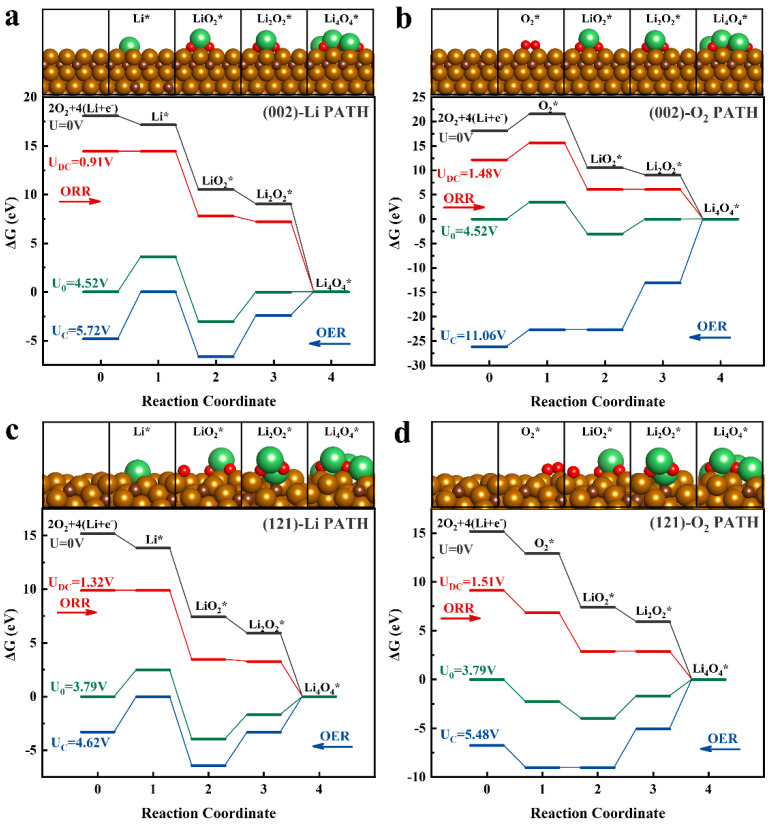
Theoretically calculated energy diagrams of the ORR and OER processes of two paths on the different surfaces: (**a**) Li PATH process on (002); (**b**) O_2_ PATH process on (002); (**c**) Li PATH process on (121); (**d**) O_2_ PATH process on (121).

The product formation and decomposition mechanisms of different crystal planes were analyzed by DFT calculations. The two main stabilized and active surfaces, namely (002) and (121) surfaces with low index and low surface energy (E_surf-(002)_ = −0.16 eV Å^−2^, E_surf-(121)_ = −0.18 eV Å^−2^), were determined through the characterization of the crystal form and microstructure of the material, and the thermodynamic reaction paths of the two surfaces were analyzed, as shown in Figure 5. Two different reaction pathways were designed, with the initial adsorbate as the difference:

Li-PATH:

(i) (Li^+^ + e^−^) → Li*
(ii) Li* + O_2_ → LiO_2_*
(iii) LiO_2_* + (Li^+^ + e^−^) → Li_2_O_2_*
(iv) Li_2_O_2_* + 2(Li^+^ + e^−^) + O_2_ → Li_4_O_4_*

O_2_-PATH:

(i) O_2_→ O_2_*
(ii) O_2_* + (Li^+^ + e^−^) → LiO_2_*
(iii) LiO_2_* + (Li^+^ + e^−^) → Li_2_O_2_*
(iv) Li_2_O_2_* + 2(Li^+^ + e^−^) + O_2_ → Li_4_O_4_*

The symbol * indicates the adsorbed species on the surface.

**Figure 6:**
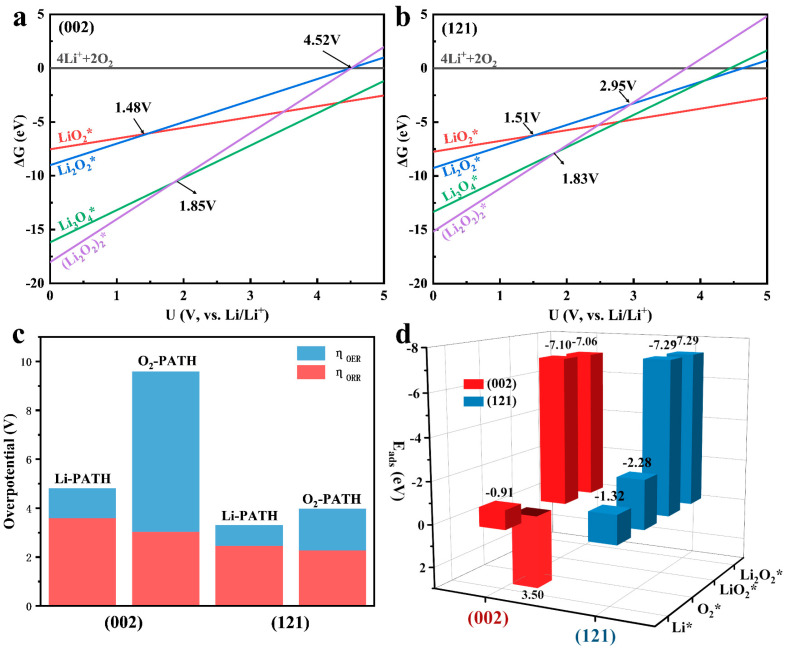
Potential-dependent phase diagram of different products for (002) (**a**) and (121) (**b**); (**c**) overpotentials of (002) and (121) in two paths; (**d**) adsorption energy (Eads) between different adsorbates and the surface of Fe_3_C.

The analysis of the four reaction paths on the two surfaces in Figure 5 shows that the ORR process is very easy to occur on account of the strong energy downhill characteristics in Li-PATH, but the steep free energy change (ΔG = 6.66 eV) of the LiO_2_ deoxidation process results in a higher energy barrier in the OER process. Similarly, the positive adsorption energy of O_2_ in the O_2_-PATH on the (002) surface (E_ads_ = 3.50 eV, Figure 6d) and the extremely high free energy change (ΔG = 11.06 eV) of the LiO_2_ delithiation process make O_2_-PATH hard to accomplish. The two paths of the (121) surface have similar characteristics to those of (002), but due to their strong adsorption of Li and O_2_ (E_ads,Li_ = –1.32 eV, E_ads,O₂_ = –2.27 eV), shown in Figure 6d, they have lower ORR and OER overpotentials. In particular, the Li-PATH of the (121) plane obtained an overpotential of 0.83 V during the OER process, which was much lower than that of other reactions, as shown in Figure 6c and Table S1. Figure 6a,b shows the potential-dependent product phase diagrams in the cathodic reaction of the two surfaces. Similarly, LiO_2_, as an intermediate product, has a higher priority in initial product formation (above 1.48 V for (002) and 1.51 V for (121)), and in subsequent product accumulation, Li_3_O_4_ (LiO_2_ and Li_2_O_2_ mixed clusters) are more easily formed (higher than 1.85 V for (002) and 1.83 V for (121)). Otherwise, the upper voltage limit for the stable accumulation of products on (002) reaches 4.52 V, which means that Li_2_O_2_ on the surface will require a higher overpotential to be decomposed. On the (121) plane, the product needs a lower overpotential to be decomposed because the upper limit of the voltage is only 2.95 V, which is the same conclusion obtained by the calculated free energy analysis.

In summary, the Fe_3_C-CF cathode exhibited outstanding cycle stability at low current density, and the discharge products were mainly Li_2−x_O_2_. The DFT calculations reveal that the Fe_3_C catalyst is beneficial for the formation of LiO_2_ and limits the conversion from LiO_2_ to Li_2_O_2_, which corresponds with the experimental result. The discharge product Li_2−x_O_2_ can provide relatively high electron and ion conduction during the ORR/OER process. As a result, we can conclude that Fe_3_C is an efficient catalyst based on the formation/decomposition of Li_2−x_O_2_. 

## 3. Materials and Methods

Synthesis of the Fe_3_C-CF: In this work, all chemicals were analytical grade and did not need further purification. The catalyst was synthesized by electrostatic spinning and high-temperature calcination in a tube furnace, as shown in Figure 1a. Briefly, dimethylformamide (DMF, 10 mL) and acetic acid (2 mL) were mixed on a magnetic rotary machine. Polyvinyl pyrrolidone (PVP, 1.5 g) was added to the mixture as a carbon source, and FeCl_2_·4H_2_O (0.795 g) was added to the above solution. The mixture was stirred for 1 h to obtain the electrospinning precursor. The electrospinning precursor was sucked into the electrospinning injector; the spinning voltage was adjusted to 13 KV, the liquid pushing speed of the electrospinning machine was controlled to 1.5 mL h^−1^, and aluminum foil paper was attached to the roller collector to collect spinning products. The collected spinning products were placed in a drying oven at a drying temperature of 50 °C. After drying for 12 h, the spinning products were transferred to a tubular furnace and heated to 600 °C, 700 °C, and 800 °C at a heating rate of 3 °C min^−1^ in an argon atmosphere. After holding for 2 h, Fe_3_C-CF samples were obtained, which were denoted Fe_3_C-600, Fe_3_C-700, and Fe_3_C-800, respectively. 

Materials characterization: The morphology of the catalyst was characterized by thermal field emission scanning electron microscopy (SEM; Hitachi, S-4800 and transmission electron microscopy (TEM; JEOL, JEM-2100F). X-ray diffraction (XRD; Rigaku D/max 2500) was used to investigate the phase composition with Cu-Kα (λ = 1.540 Å) radiation over a voltage of 36 kV and a current of 20 mA. The surface elemental composition and valence of the catalyst were tested by X-ray photoelectron spectroscopy (XPS; ESCALAB 250). Raman spectroscopy was used to measure the degree of graphitization using a Via Reflex instrument with laser excitation at 532 nm. The evolution rate profiles of different gas species were monitored by an in situ differential electrochemical mass spectrometry (DEMS) system (i-DEMS 100) using secondary electron multiplier mode with an EI-70 ev ion source. The specific surface area and pore size distribution of the material were determined by N_2_ adsorption and desorption isotherms through Brunauer–Emmett–Teller (BET) analysis. 

Assembly of Li-O_2_ batteries and electrochemical measurements: The oxygen electrode was obtained by coating a slurry mixture of 40 wt% Fe_3_C-CF, 40 wt% CNT, and 20 wt% PTFE on carbon paper. The average Fe_3_C-CF loading mass on each carbon (diameter 19 mm) paper was 0.4 mg, and the specific capacity was calculated based on its total mass. The positive plate was dried in a vacuum drying oven at 120 °C for 12 h. All the assembly processes of the cells were completed in a glove box under an argon-protected atmosphere. The electrolyte for the battery was composed of 1 M lithium nitrate (LiNO_3_) in dimethyl sulfoxide (DMSO). CT2001A was used to test the cyclic performance and capacity of the battery at a voltage of 2.0 to 4.5 V. The cyclic voltammogram (CV) test was operated through an electrochemical workstation (CHI660D) with a sweep speed condition of 0.15 mV s^−1^, a minimum voltage of 2.35 V, and a maximum voltage of 4.35 V. The AC amplitude for the electrochemical impedance spectroscopy (EIS) test was 5 mV, and the frequency range of the EIS measurements was 0.01 Hz to 100,000 Hz. 

DFT Calculations: DFT calculations were performed using the Vienna ab initio Simulation Package (VASP) based on DFT. The projector-augmented wave was used to describe the electron–ion interaction. The Perdew–Burke–Ernzerh (PBE) function of the ( generalized gradient approximation GGA) was used to analyze the electron exchange and correlation energies. The kinetic energy cutoff for the planewave expansion was set to 350 eV, which can provide sufficient computational accuracy. The convergence criterion for the total energy was 10^−5^ eV. All atomic positions were relaxed until the force of each atom was reduced to less than 0.02 eV Å^−1^. The length of each side of the vacuum layer was considered to be 15 Å. 

## 4. Conclusions

To improve the cycle life, capacity performance, and rate performance of lithium-oxygen batteries as much as possible, Fe_3_C particles were uniformly loaded onto carbon fibers with a three-dimensional network structure by a two-step synthesis method. The Fe_3_C-700 cathode exhibited excellent electrochemical performance due to its rich porous structure and high degree of graphitization. This Fe_3_C-700 cathode delivered an outstanding specific capacity of 17,653.1 mAh g^−1^ and an excellent reversibility of 263 cycles at a fixed capacity of 600 mAh g^−1^. The outstanding electrochemical performance of the Fe_3_C-CF electrode originated from the synergistic effect of the intrinsically good catalytic ability of Fe_3_C and the high electronic conductivity of carbon fibers. Furthermore, the in situ obtained 3D mesoporous structure of Fe_3_C-CF is strong enough to prevent volume expansion in the catalytic process and benefit the deposition of the discharge product Li_2−x_O_2_ and the transfer of oxygen and electrolyte. The small particle size of Fe_3_C and its uniform distribution are also conducive to forming a thin discharge product film layer, which enhances the electronic conductivity, postpones electrode passivation, and thus prolongs the cycle life. DFT calculations show that the strong adsorption of Li and O_2_ on the (121) crystal plane reduces the reaction energy barriers of the ORR and OER processes and achieves an OER overpotential of 0.83 V through Li-PATH. This work will provide a reference for the application of a Fe_3_C-based catalyst cathode that possesses excellent catalytic performance in LOBs.

## Data Availability

Not applicable.

## Supplementary Materials

The following supporting information can be downloaded at: https://www.mdpi.com/article/10.3390/molecules28145597/s1, Figure S1: SEM images of Fe_3_C-600 (**a**) and Fe_3_C-800 (**b**) after carbonization; Figure S2: SEM images and elemental mapping of Fe and C in Fe_3_C-600 (**a**) and Fe3C-800 (**b**); Figure S3: Line SEM images and elemental mapping of the Fe_3_C-700; Figure S4: Nitrogen adsorption/desorption isotherms of Fe_3_C-600 (**a**) and Fe_3_C-800 (**b**); Pore size distribution of Fe_3_C-600 (**c**) and Fe_3_C-800 (**d**); Figure S5: EIS spectra of Fe_3_C-CF electrodes before and after CV; Figure S6: Galvanostatic discharge/charge curves of Fe_3_C-700 within an upper-limited specific capacity of 600 mAh g^-1^ at a current density of 200 mA g^−1^ (**a**), 800 mA g^−1^ (**b**), and 1000 mA g^−1^ (**c**); Figure S7: Electrochemical impedance spectra of Fe_3_C-700 at different stages; Figure S8: (**a**) In situ DEMS curves of the Fe_3_C-700 catalyst, which were collected during the charging process with a specific capacity of 600 mAh g^−1^ at a current density of 500 mA g^−1^; (**b**) High-resolution XPS spectra of the C 1s core level of the Fe_3_C-700 electrode after the 50th recharge; Table S1: Overpotentials of the ORR and OER processes of two paths on the different surfaces; Table S2: Adsorption energy (E_ads_) between different adsorbates and the surface of Fe_3_C.

## Sample Availability

Not available.
