# Supplementary material for: Efficient Fe3C-CF Cathode Catalyst Based on the Formation/Decomposition of Li2−xO2 for Li-O2 Batteries
Source: Molecules. 2023 Jul 24;28(14):5597. doi: 10.3390/molecules28145597 (PMC10385974; doi:10.3390/molecules28145597)
Supplement: Supplementary file 1 [file molecules-28-05597-s001.zip › molecules-2502687-supplementary.pdf]

molecules

Supporting Information

Efficient Fe_3C-CF cathode catalyst based on the formation/decomposition of $Li_{2-x}O_2$ for Li-O_2 batteries

Guanyu Yi [1,†], Gaoyang Li [2,†], Shuhuai Jiang [1], Guoliang Zhang [2], Liang Guo [2], Xiuqi Zhang [2], Zhongkui Zhao [1,*] Zhongping Zou [1], Hailong Ma [1], Xiaojiao Fu [1], Yan Liu [1] and Feng Dang [2,*]

[1] School of Materials Science and Engineering, Shandong jianzhu University, Jinan 250101, China;
[2] Key Laboratory for Liquid-Solid Structural Evolution and Processing of Materials (Ministry of Education), Shandong University, Jinan 250061, China;
* Correspondence: yiguanyu@sdjzu.edu.cn (G.Y.); jorezhk@sdjzu.edu.cn (Z.Z.); dangfeng@sdu.edu.cn (F.D.)
† These authors contributed equally to this work.

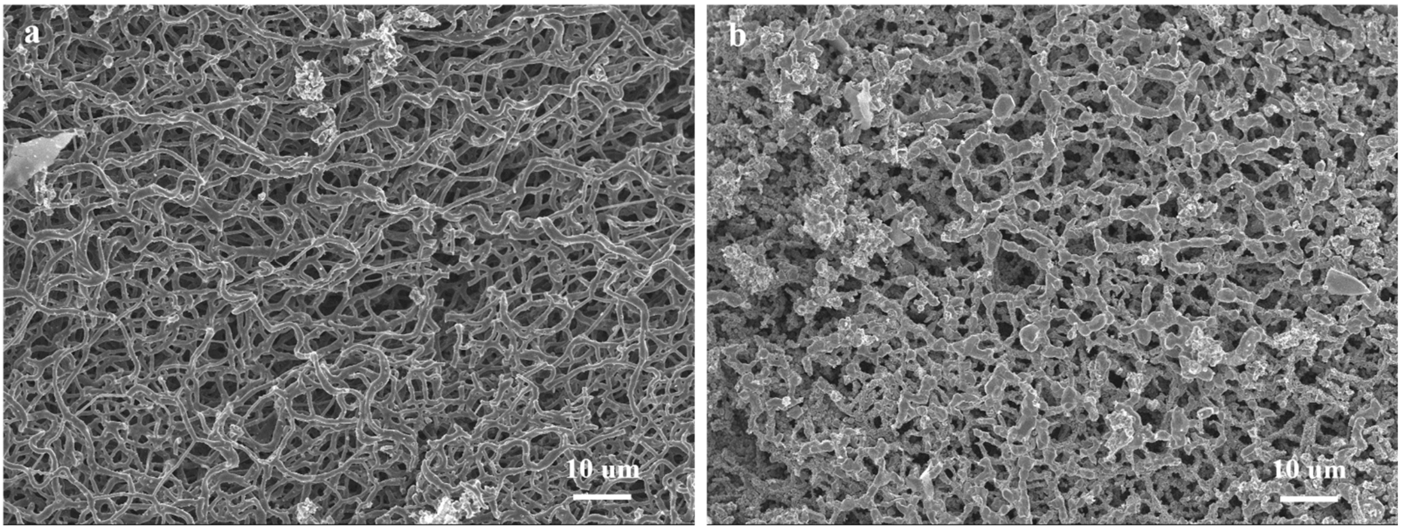

Figure S1. The SEM images of Fe_3C-600 (a) and Fe_3C-800 (b) after carbonization.

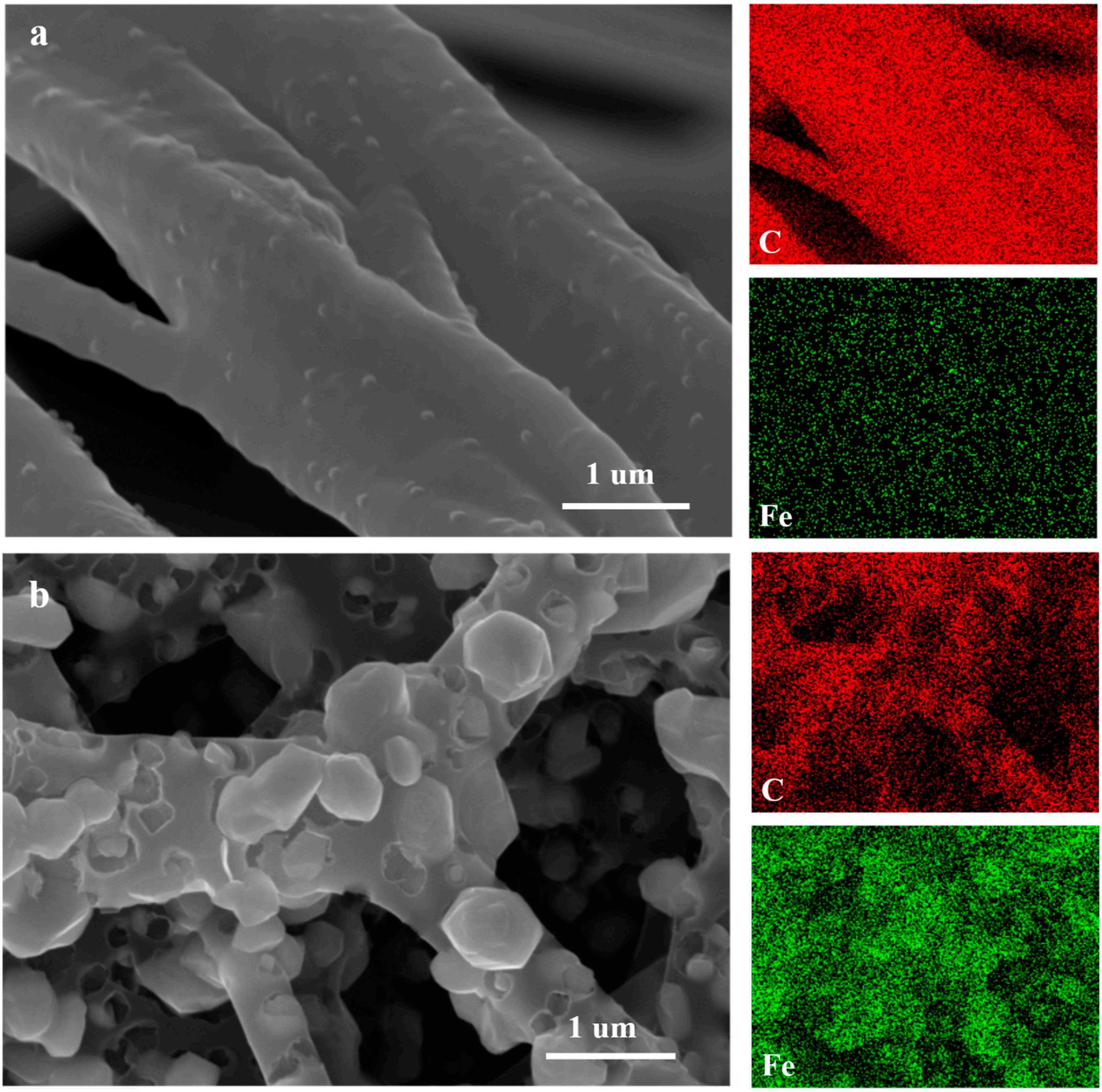

Figure S2. SEM images and elemental mapping of Fe and C in Fe_3C-600 (a), and Fe_3C-800 (b).

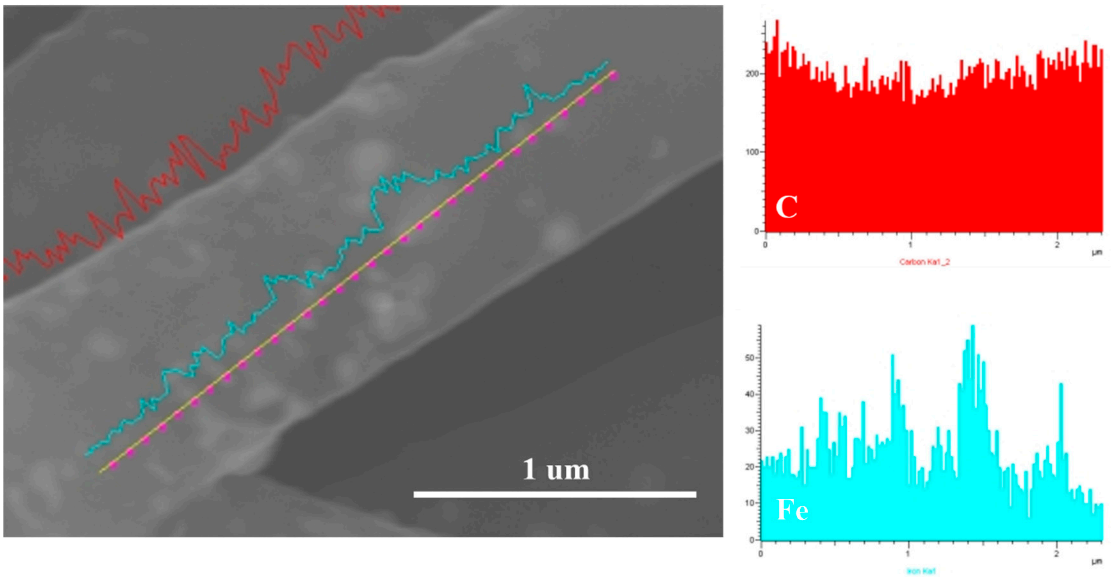

Figure S3. Line SEM images and elemental mapping of the Fe_3C-700.

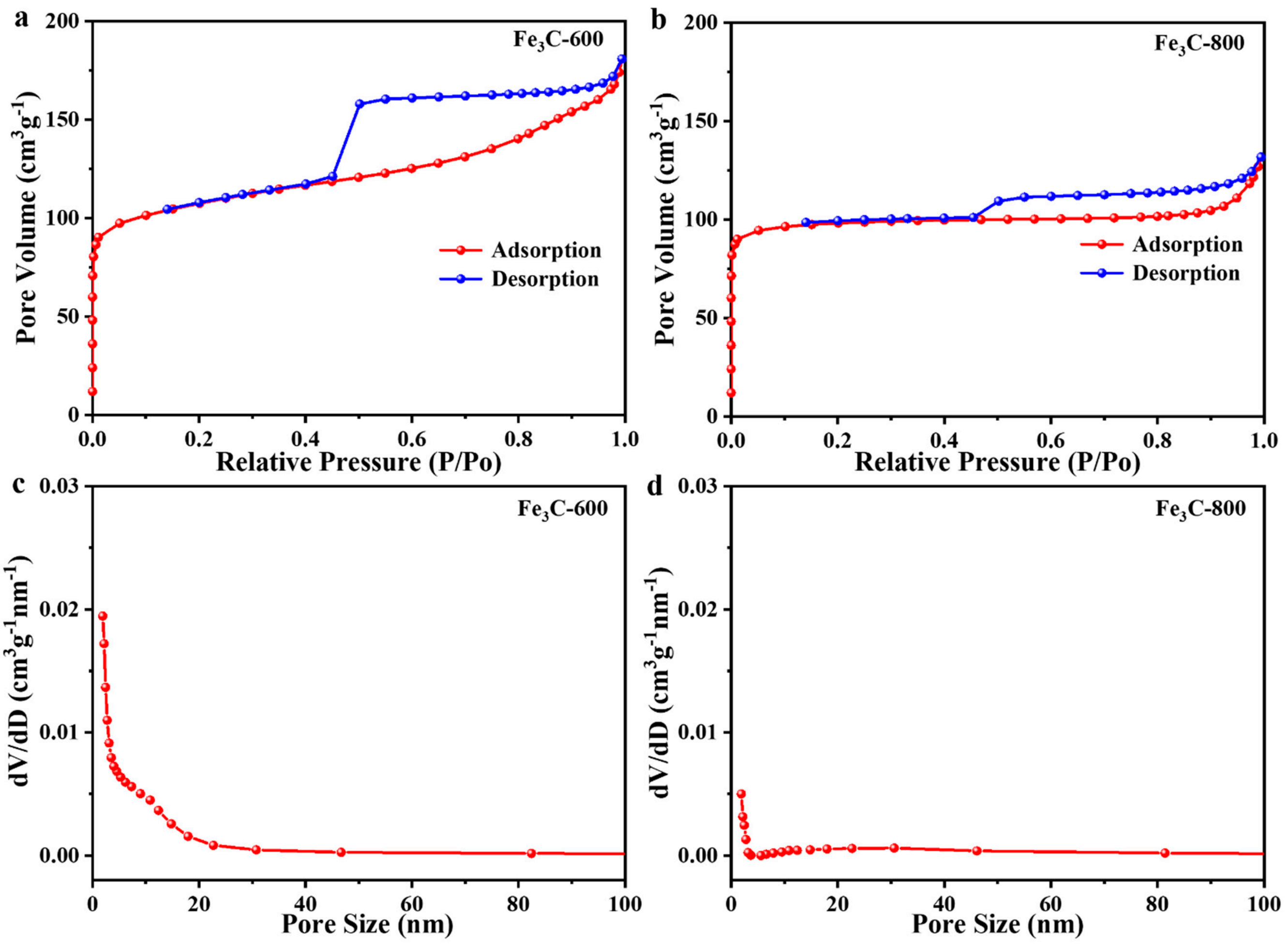

Figure S4. Nitrogen adsorption/desorption isotherms of Fe_3C-600 (a) and Fe_3C-800 (b); The pore size distribution of Fe_3C-600 (c) and Fe_3C-800 (d).

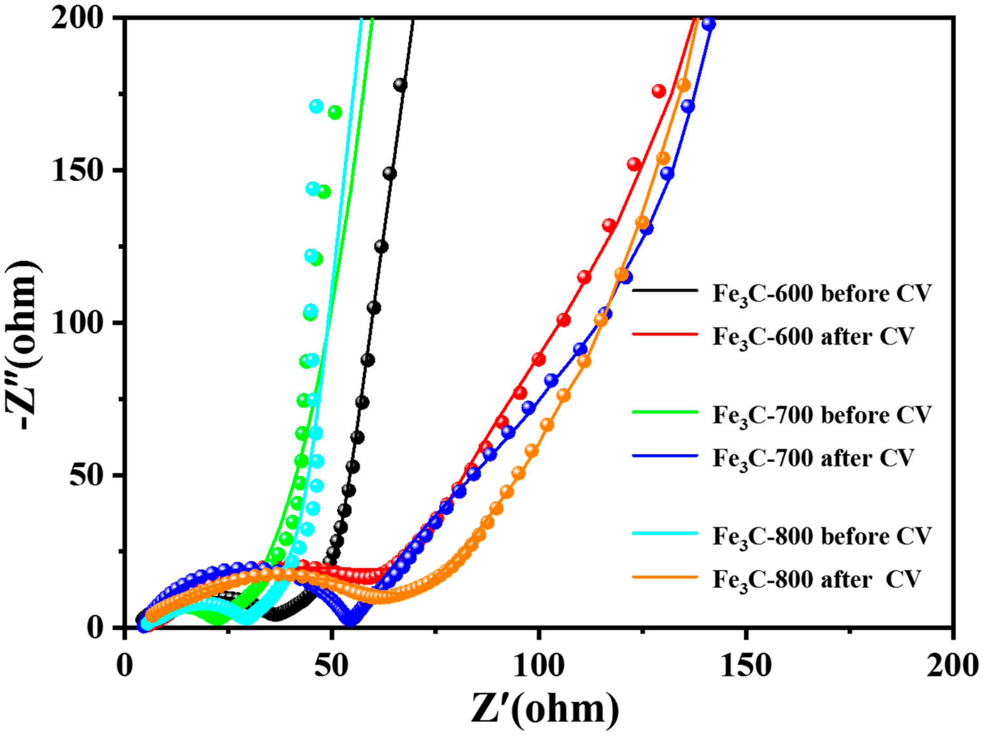

Figure S5. EIS spectrums of Fe_3C-CF electrodes before and after CV.

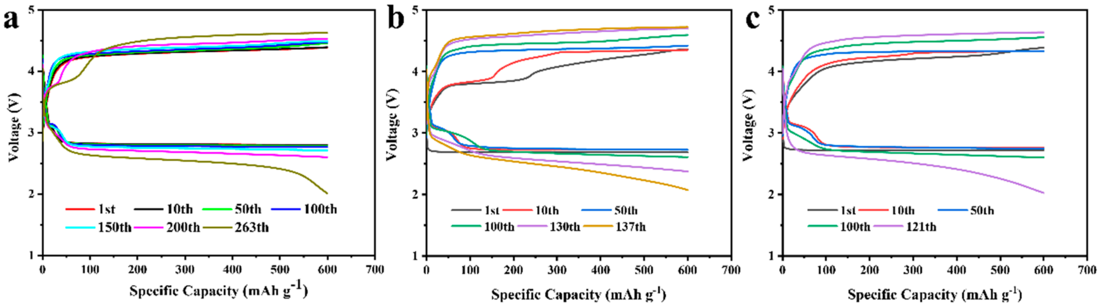

Figure S6. Galvanostatic discharge/charge curves of Fe_3C-700 within an upper-limited specific capacity of 600 mAh g^{-1} at a current density of 200 mA g^{-1}(a), 800 mA g^{-1}(b), 1000 mA g^{-1}(c).

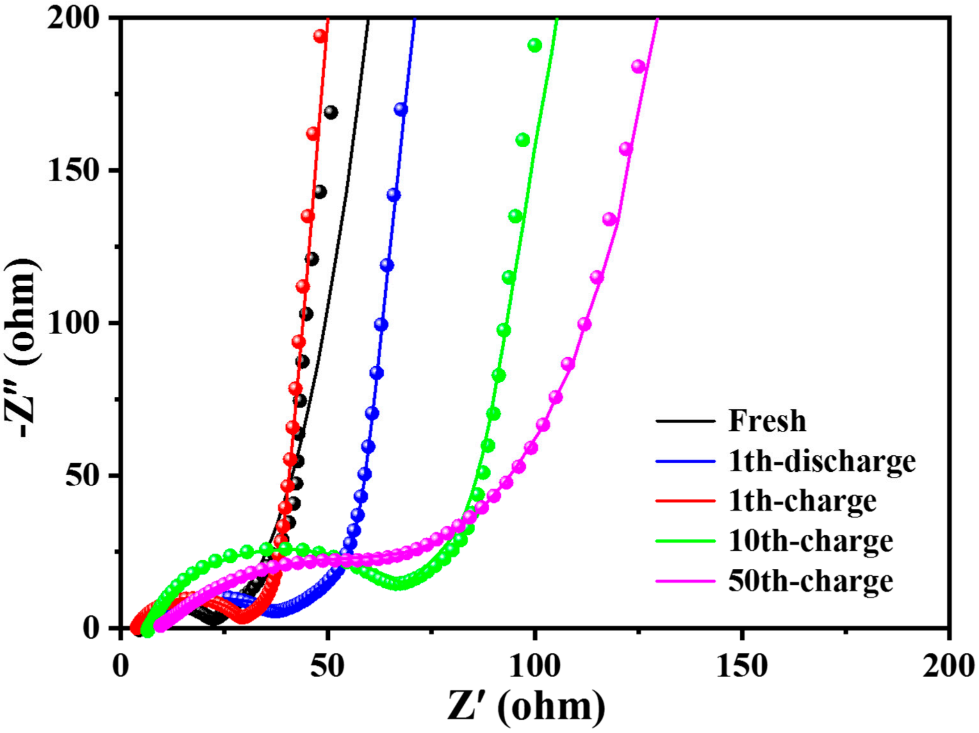

Figure S7. Electrochemical impedance spectra of Fe_3C-700 at different stages.

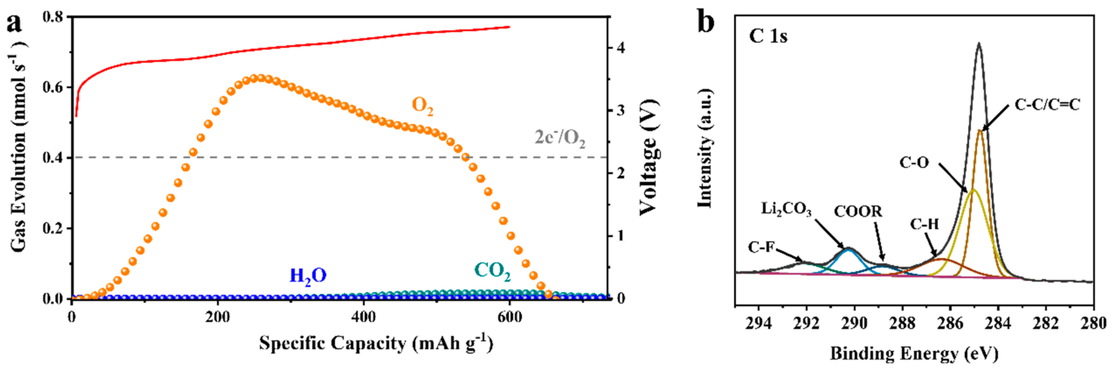

Figure S8. (a) In-situ DEMS curves of the Fe_3C-700 catalyst, which were collected during the charging process with a specific capacity of 600 mAh g^{-1}, at a current density of 500 mA g^{-1}; (b) High-resolution XPS spectra of C 1s core level of the Fe_3C-700 electrode after 50th recharged.

Table S1. Overpotentials of the ORR and OER processes of two paths on the different surfaces.

Copyright: © 2023 by the authors. Licensee MDPI, Basel, Switzerland. This article is an open access article distributed under the terms and conditions of the Creative Commons Attribution (CC BY) license (https://creativecommons.org/licenses/by/4.0/).

	**(002) Li-PATH**	**(002) O_2-PATH**	**(121) Li-PATH**	**(121) O_2-PATH**
**U_{DC}(V)**	0.91	1.48	1.32	1.51
**U_0(V)**	4.52	4.52	3.79	3.79
**U_C(V)**	5.72	11.06	4.62	5.48
**η_{ORR}(V)**	3.61	3.04	2.47	2.28
**η_{OER}(V)**	1.20	6.54	0.83	1.69
**η_{TOT}(V)**	4.81	9.58	3.30	3.97

Table S2. Adsorption energy (E_{ads}) between different adsorbates and surface of Fe_3C.

E_{ads}(eV)	(002)	(121)
**Li**	-0.90897	-1.32169
**O_2**	3.49652	-2.27596
**LiO_2**	-7.09868	-7.29455
**Li_2O_2**	-7.06259	-7.29278

Disclaimer/Publisher's Note: The statements, opinions and data contained in all publications are solely those of the individual author(s) and contributor(s) and not of MDPI and/or the editor(s). MDPI and/or the editor(s) disclaim responsibility for any injury to people or property resulting from any ideas, methods, instructions or products referred to in the content.
